# Stress within a Restricted Time Window Selectively Affects the Persistence of Long-Term Memory

**DOI:** 10.1371/journal.pone.0059075

**Published:** 2013-03-27

**Authors:** Chang Yang, Jian-Feng Liu, Bai-Sheng Chai, Qin Fang, Ning Chai, Li-Yan Zhao, Yan-Xue Xue, Yi-Xiao Luo, Min Jian, Ying Han, Hai-Shui Shi, Lin Lu, Ping Wu, Ji-Shi Wang

**Affiliations:** 1 Affiliated Hospital and School of Pharmacy of Guiyang Medical University, Guiyang, China; 2 National Institute on Drug Dependence, Peking University, Beijing, China; 3 Institute of Mental Health, Hebei Medical University, Shijiazhuang, China; 4 Hebei Brain Ageing and Cognitive Neuroscience Laboratory, Hebei Medical University, Shijiazhuang, China; 5 Department of Biochemistry and Molecular Biology, Basic Medical College, Hebei Medical University, Shijiazhuang, China; Université Pierre et Marie Curie, France

## Abstract

The effects of stress on emotional memory are distinct and depend on the stages of memory. Memory undergoes consolidation and reconsolidation after acquisition and retrieval, respectively. Stress facilitates the consolidation but disrupts the reconsolidation of emotional memory. Previous research on the effects of stress on memory have focused on long-term memory (LTM) formation (tested 24 h later), but the effects of stress on the persistence of LTM (tested at least 1 week later) are unclear. Recent findings indicated that the persistence of LTM requires late-phase protein synthesis in the dorsal hippocampus. The present study investigated the effect of stress (i.e., cold water stress) during the late phase after the acquisition and retrieval of contextual fear memory in rats. We found that stress and corticosterone administration during the late phase (12 h) after acquisition, referred to as late consolidation, selectively enhanced the persistence of LTM, whereas stress during the late phase (12 h) after retrieval, referred to as late reconsolidation, selectively disrupted the restabilized persistence of LTM. Moreover, the effects of stress on the persistence of LTM were blocked by the corticosterone synthesis inhibitor metyrapone, which was administered before stress, suggesting that the glucocorticoid system is involved in the effects of stress on the persistence of LTM. We conclude that stress within a restricted time window after acquisition or retrieval selectively affects the persistence of LTM and depends on the glucocorticoid system.

## Introduction

Newly learned information goes through several stages to form long-term memory (LTM) [1]–[4]. In the classical contextual fear conditioning model, a contextual conditioned stimulus (CS) is associated with a fearful unconditioned stimulus (US) [5]–[7]. After conditioning, the CS-US association memory is labile and undergoes the consolidation process [1]. Consolidation occurs immediately after conditioning but persists for several hours, days, or even months after training, after which memory is stably stored in the brain [8]–[10]. Consolidated memory reenters an unstable state after retrieval (i.e., exposure to the CS or US), regardless of the timing of the exposure or age of the memory, which requires the reconsolidation process to be stably stored again [7], [11]–[16]. Memory within the consolidation and reconsolidation time windows can be modified, updated, or disrupted using pharmacological or behavioral manipulations [11], [17]–[22].

Recently, a late consolidation process was proposed, suggesting that memory after learning within a delayed time window was also labile [23]–[26]. After learning, a delayed protein synthesis process is critical for the persistence but not formation of LTM [23], [24]. Several types of molecules have been shown to participate in the late consolidation process, and the modulation of their activity can either destroy or enhance the persistence of LTM [24], [25], [27], [28]. Previous research identified a system consolidation phenomenon to explain the persistence of LTM, suggesting that a memory that was recently stored in the hippocampus will ultimately transfer to the neocortex to form a remote memory [4]. Late consolidation was suggested to function as a bridge between cellular and system consolidation [28].

Memory consolidation and reconsolidation can be modulated by environment stimuli, such as stress. Stress distinctly affects memory consolidation and reconsolidation, which has been demonstrated in both humans and animals [29]–[36]. When stress was administered within the consolidation stage after learning, subsequent memory performance was enhanced [32]–[35]. But when stress was administered within the reconsolidation stage after retrieval, the original memory was disrupted [30], [31], [37]. The differential effects of stress on different memory stages can be explained by the different timing of the actions of stress hormones, especially norepinephrine and corticosteroids [29], [30], [37]–[40]. Moreover, although many signaling pathways that are engaged in consolidation play a role in reconsolidation, a distinct mechanism has also been proposed [12] that might be another explanation for the differential effects of stress on these two similar memory processes. Even if the effects of stress on memory consolidation and reconsolidation were clearly identified in previous studies, such studies focused only on a short time window after learning or retrieval. The effects of stress within the delayed time window after learning and retrieval are still unclear [41].

In the present study, we determined the effect of stress within a delayed time window after learning and retrieval on subsequent memory performance. First, we tested the effect of the cold swim stress and corticosterone administration at different time points after contextual fear memory acquisition on memory performance. Second, we tested the effect of cold swim stress and corticosterone administration at different time points after contextual fear memory retrieval on subsequent memory performance. Third, we investigated whether the effects of stress at different time points after contextual fear memory acquisition and retrieval depend on corticosterone synthesis.

## Results

### Stress and corticosterone administration during the late phase after fear conditioning selectively enhanced the persistence of LTM

**Figure 1:**
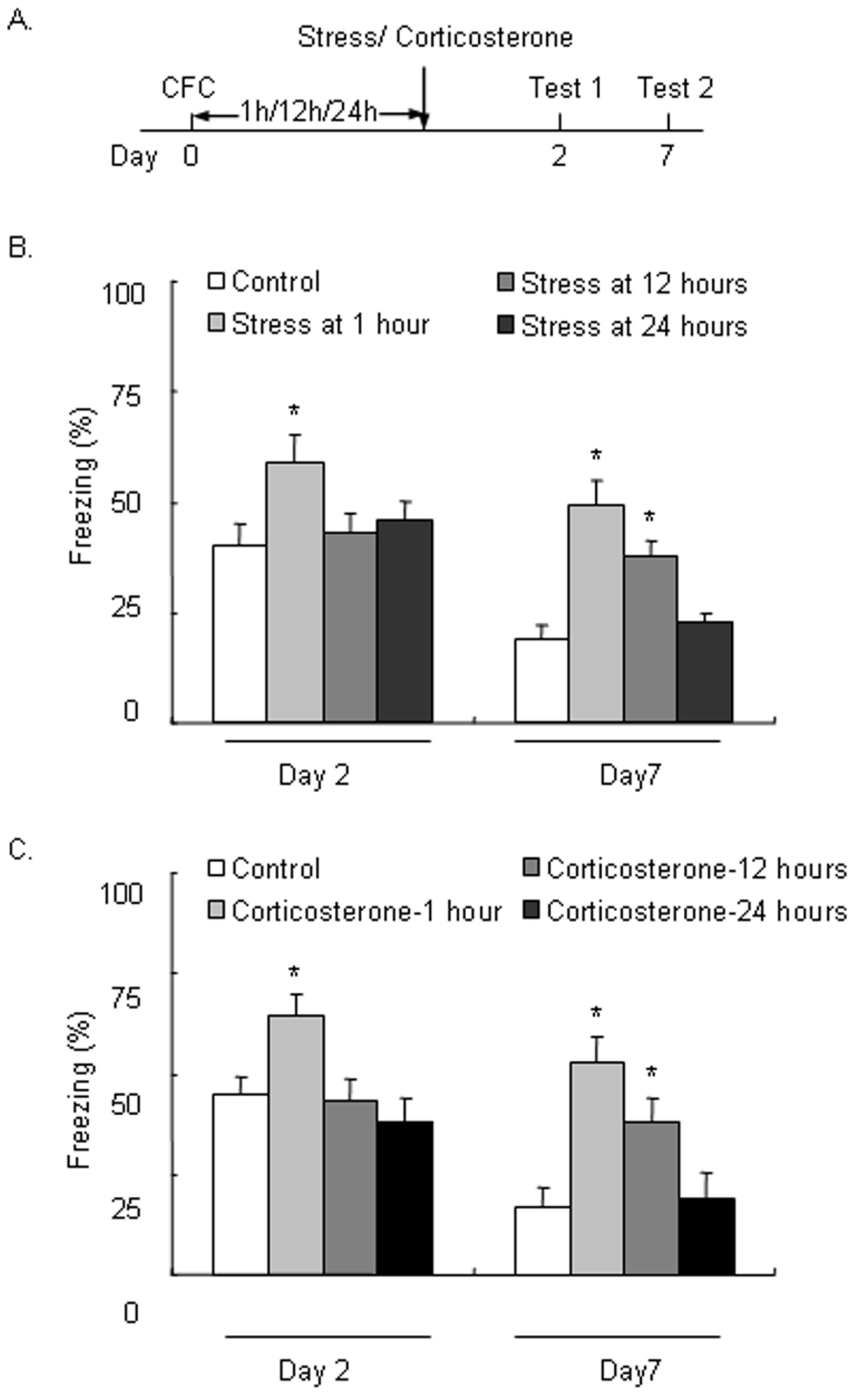
Stress and corticosterone administered 12 h after fear conditioning selectively enhanced the persistence but not formation of long-term memory. (A) Rats were exposed to cold water stress or administered corticosterone 1, 12, or 24 h after weak contextual fear conditioning (CFC) and then tested for long-term memory (LTM) on day 2 and day 7. (B) Stress exposure 1 h after CFC enhanced LTM formation tested on day 2, whereas stress exposure 12 h after CFC selectively enhanced the persistence of LTM tested on day 7 but did not affect LTM formation tested on day 2. (C) Corticosterone administration 1 h after CFC enhanced LTM formation tested on day 2, whereas corticosterone administration 12 h after CFC selectively enhanced the persistence of LTM tested on day 7 but did not affect LTM formation tested on day 2. The data are expressed as mean ± SEM (*n* = 6–9 per group). *p<0.05, compared with Control group.

In Experiment 1, we first tested the effect of stress at different time points (1, 12, and 24 h) after fear conditioning on LTM. A repeated-measures analysis of variance (ANOVA) was conducted to analyze the time spent freezing in rats 2 and 7 days after fear conditioning, with Test day (day 2 and day 7) as the within-subjects factor and Stress (No stress, stress at 1 h, stress at 12 h, and stress at 24 h) as the between-subjects factor. The analysis showed significant effects of Test day (*F*_1,31_ = 35.590, *p*<0.05) and Stress (*F*_3,31_ = 8.975, *p*<0.05) and a significant Test day × Stress interaction (*F*_3,31_ = 3.122, *p*<0.05). The *post hoc* analysis showed that stress administered 1 h after fear conditioning enhanced the time spent freezing in the tests on days 2 and 7 (both *p*<0.05), but stress administered 12 h after fear conditioning only enhanced the time spent freezing in the test on day 7 (*p*<0.05) but not day 2 (*p*>0.05; Fig. 1B).

We then tested the effect of the stress hormone corticosterone at different time points (1, 12, and 24 h) after fear conditioning on LTM. A repeated-measures ANOVA was conducted to analyze the time spent freezing 2 days and 7 days after fear conditioning, with Test day (day 2 and day 7) as the within-subjects factor and Corticosterone (No corticosterone, corticosterone at 1 h, corticosterone at 12 h, and corticosterone at 24 h) as the between-subjects factor. The analysis showed significant effects of Test day (*F*_1,20_ = 40.376, *p*<0.05) and Corticosterone (*F*_3,31_ = 8.482, *p*<0.05) and a significant Test day × Corticosterone interaction (*F*_3,31_ = 3.720, *p*<0.05). The *post hoc* analysis showed that corticosterone administered 1 h after fear conditioning enhanced the time spent freezing in the tests on days 2 and 7 (both *p*<0.05), but corticosterone administered 12 h after fear conditioning only enhanced the time spent freezing in the test on day 7 (*p*<0.05), leaving the time spent freezing on day 2 intact (*p*>0.05; Fig. 1C). These results indicate that both stress and corticosterone enhanced the formation of LTM when administered 1 h after fear conditioning but selectively enhanced the persistence of LTM when administered 12 h after fear conditioning. The enhancement of the formation or persistence of LTM by stress might be mediated by the stress hormone corticosterone.

### Metyrapone blocked the enhancement of LTM formation and persistence induced by stress after fear conditioning

**Figure 2:**
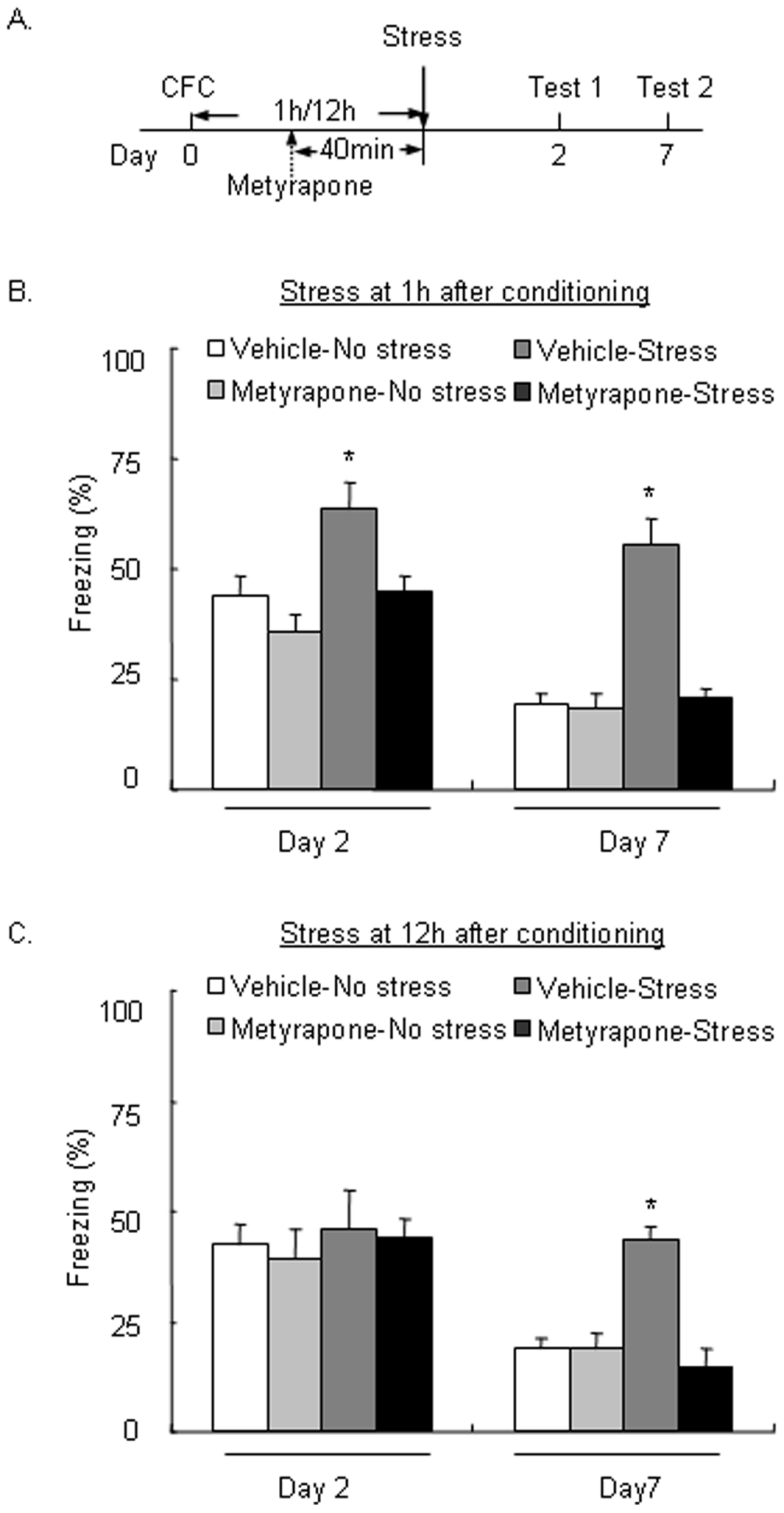
Metyrapone blocked the enhancement of long-term memory formation or persistence induced by stress after fear conditioning. (A) Rats were pretreated with the corticosterone synthesis inhibitor metyrapone 40 min before exposure to cold water stress 1 or 12 h after weak contextual fear conditioning (CFC) and then tested for long-term memory (LTM) on day 2 and day 7. (B) Metyrapone blocked the effects of stress 1 h after CFC on LTM formation on day 2 and reduced the persistence of LTM tested on day 7. (C) Metyrapone blocked the effects of stress 12 h after CFC on the persistence of LTM tested on day 7, leaving LTM formation tested on day 2 intact. The data are expressed as mean ± SEM (*n* = 6 per group). *p<0.05, compared with Vehicle-No stress group.

In Experiment 2, we tested whether a corticosterone synthesis inhibitor can block the effect of stress after fear conditioning on LTM. The rats were pretreated with metyrapone 40 min before stress exposure. A two-way repeated-measures ANOVA, with Test day (day 2 and day 7) as the within-subjects factor and Metyrapone (0 and 40 mg/kg) and Stress (no stress, stress at 1 h, and stress at 12 h) as the between-subjects factors, was used to analyze the time spent freezing in rats that were exposed to stress 1 or 12 h after fear conditioning, respectively. For stress exposure 1 h after fear conditioning, the analysis showed significant effects of Test day (*F*_1,20_ = 44.039, *p*<0.05), Stress (*F*_1,20_ = 33.427, *p*<0.05), and Metyrapone (*F*_1,20_ = 29.103, *p*<0.05) and significant Stress × Metyrapone (*F*_1,20_ = 14.313, *p*<0.05) and Test day × Stress × Metyrapone (*F*_1,20_ = 4.423, *p*<0.05) interactions. The *post hoc* analysis showed that metyrapone blocked the enhancement of LTM formation induced by stress 1 h after fear conditioning (*p*<0.05; Fig. 2B). For stress exposure 12 h after fear conditioning, the analysis showed significant effects of Test day (*F*_1,20_ = 40.013, *p*<0.05), Stress (*F*_1,20_ = 6.854, *p*<0.05), and Metyrapone (*F*_1,20_ = 7.998, *p*<0.05) and significant Stress × Metyrapone (*F*_1,20_ = 4.693, *p*<0.05) and Test day × Stress × Metyrapone (*F*_1,20_ = 4.724, *p*<0.05) interactions. The *post hoc* analysis showed that metyrapone blocked the enhancement of LTM persistence induced by stress 12 h after fear conditioning (*p*<0.05; Fig. 2C). These results indicate that the improvement of LTM formation and persistence induced by stress after fear conditioning required corticosterone synthesis.

### Stress and corticosterone during the late phase after memory reactivation selectively disrupted the persistence of LTM

**Figure 3:**
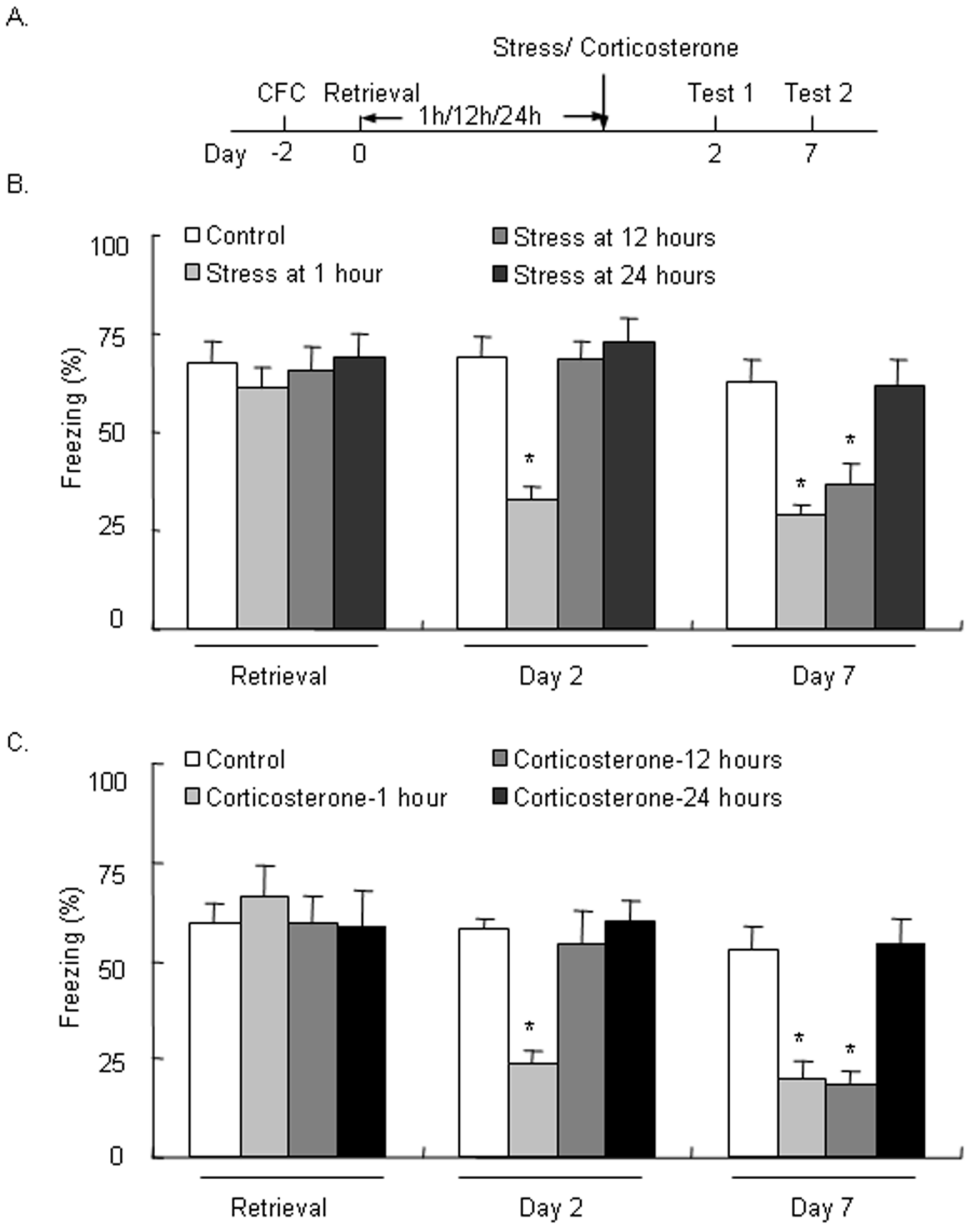
Stress and corticosterone administered 12 h after fear memory retrieval selectively impaired the persistence but not formation of long-term memory. (A) Rats were exposed to cold water stress or administered corticosterone 1, 12, or 24 h after retrieval of the memory of strong contextual fear conditioning (CFC) and then tested for long-term memory (LTM) on day 2 and day 7. (B) Stress exposure 1 h after CFC retrieval enhanced LTM reformation tested on day 2, whereas stress exposure 12 h after CFC retrieval selectively enhanced the persistence of LTM tested on day 7 but did not affect LTM reformation tested on day 2. (C) Corticosterone administration 1 h after CFC retrieval enhanced LTM formation tested on day 2, whereas corticosterone administration 12 h after CFC retrieval selectively enhanced the persistence of LTM tested on day 7 but did not affect LTM reformation tested on day 2. The data are expressed as mean ± SEM (*n* = 6–8 per group). *p<0.05, compared with Control group.

In Experiment 3, we tested the effect of stress at different time points after fear memory retrieval on LTM. Memory retrieval was not significantly different among groups (both *p*>0.05; Fig. 3B, C). A repeated-measures ANOVA, with Test day (day 2 and day 7) as the within-subjects factor and Stress (No stress, stress at 1 h, stress at 12 h, and stress at 24 h) as the between-subjects factor, was conducted to analyze the time spent freezing on days 2 and 7 after fear memory retrieval. The analysis showed significant effects of Test day (*F*_1,32_ = 13.961, *p*<0.05) and Stress (*F*_3,32_ = 23.435, *p*<0.05) and a significant Test × Stress interaction (*F*_3,32_ = 2.962, *p*<0.05). The *post hoc* analysis showed that stress given 1 h after fear memory retrieval decreased the time spent freezing on days 2 and 7 (both *p*<0.05), whereas stress given 12 h after fear memory retrieval only decreased freezing time on day 7 (*p*<0.05) but not day 2 (*p*>0.05; Fig. 3B).

We then tested the effect of the stress hormone corticosterone at different time points (1, 12, and 24 h) after fear memory retrieval on LTM. A repeated-measures ANOVA was conducted to analyze the time spent freezing 2 days and 7 days after fear memory retrieval, with Test day (day 2 and day 7) as the within-subjects factor and Corticosterone (No corticosterone, corticosterone at 1 h, corticosterone at 12 h, and corticosterone at 24 h) as the between-subjects factor. The analysis showed significant effects of Test day (*F*_1,20_ = 8.465, *p*<0.05) and Corticosterone (*F*_3,20_ = 24.017, *p*<0.05) and a significant Test × Corticosterone interaction (*F*_3,20_ = 3.196, *p*<0.05). The *post hoc* analysis showed that corticosterone administered 1 h after fear memory retrieval decreased the time spent freezing on days 2 and 7 (both *p*<0.05), but corticosterone administered 12 h after fear memory retrieval only decreased the time spent freezing on day 7 (*p*<0.05), leaving the time spent freezing on day 2 intact (*p*>0.05; Fig. 3C). These results indicate that both stress and corticosterone disrupted the restabilization of LTM when administered 1 h after fear memory retrieval but selectively disrupted the persistence of LTM when administered 12 h after fear conditioning. The disruption of the restabilization and persistence of LTM by stress might be mediated by the stress hormone corticosterone.

### Metyrapone blocked the impairment of LTM formation and persistence induced by stress after fear retrieval

**Figure 4:**
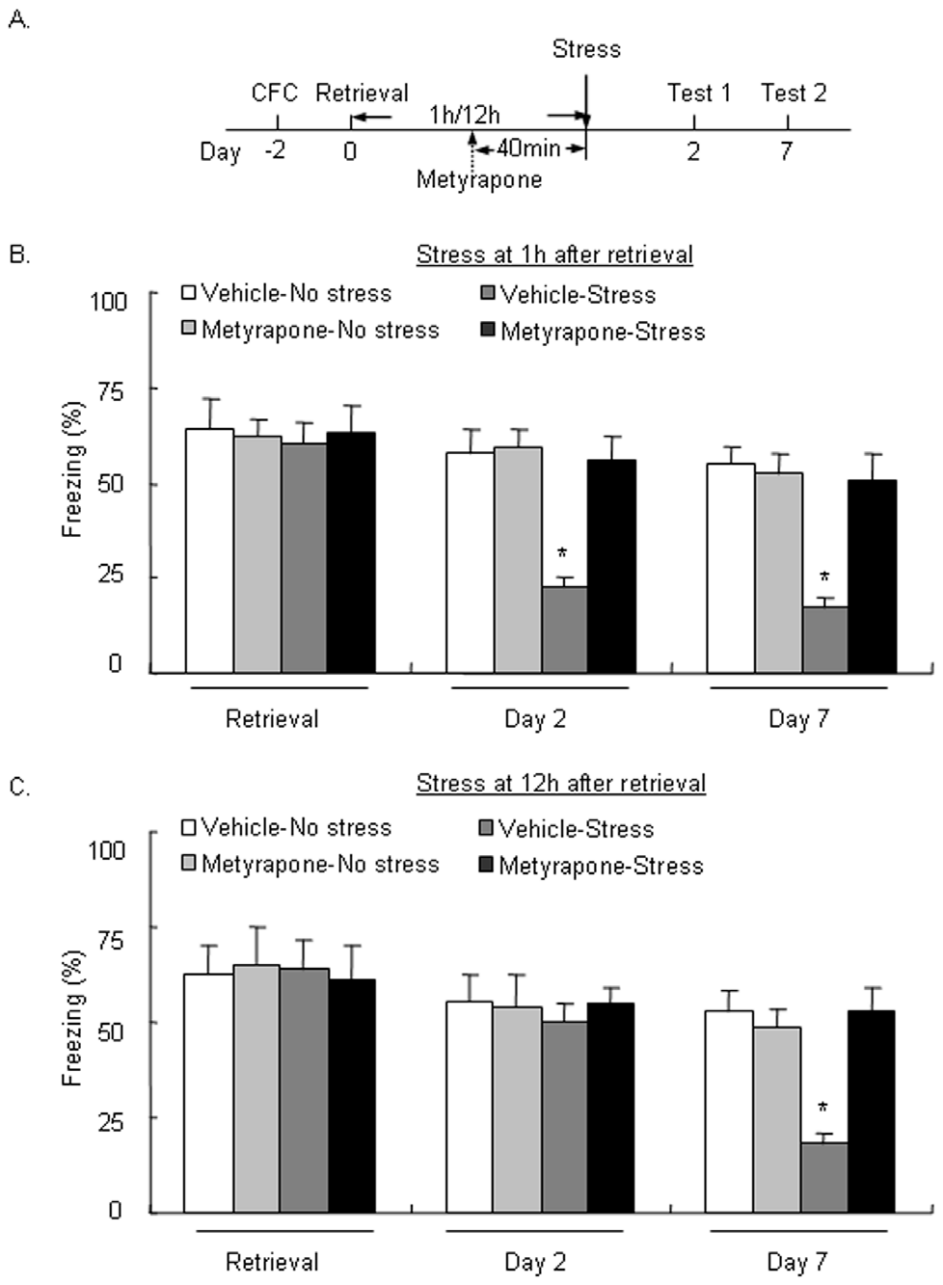
Metyrapone blocked the impairment of long-term memory formation and persistence induced by stress after fear memory retrieval. (A) Rats were pretreated with the corticosterone synthesis inhibitor metyrapone 40 min before exposure to cold water stress 1 or 12 h after retrieval of the memory of strong contextual fear conditioning (CFC) and then tested for long-term memory (LTM) on day 2 and day 7. (B) Metyrapone blocked the effects of stress 12 h after CFC retrieval on LTM formation on day 2 and decreased the persistence of LTM tested on day 7. (C) Metyrapone blocked the effects of stress 12 h after CFC retrieval on the persistence of LTM tested on day 7 but did not affect LTM formation tested on day 2. The data are expressed as mean ± SEM (*n* = 6 per group). *p<0.05, compared with Vehicle-No stress group.

In Experiment 4, we tested whether a corticosterone synthesis inhibitor can block the effect of stress after fear memory retrieval on LTM. The rats were pretreated with metyrapone 40 min before stress exposure. Memory retrieval was not significantly different among groups (both *p*>0.05; Fig. 4B, C). A two-way repeated-measures ANOVA, with Test day (day 2 and day 7) as the within-subjects factor and Metyrapone (0 and 40 mg/kg) and Stress (no stress, stress at 1 h, and stress at 12 h) as the between-subjects factors, was used to analyze the time spent freezing in rats that were exposed to stress 1 or 12 h after fear memory retrieval, respectively. For stress exposure 1 h after fear memory retrieval, the analysis showed significant effects of Stress (*F*_1,20_ = 21.834, *p*<0.05) and Metyrapone (*F*_1,20_ = 15.999, *p*<0.05) and a significant Stress × Metyrapone interaction (*F*_1,20_ = 16.542, *p*<0.05) but no significant effect of Test day (*F*_1,20_ = 3.098, *p*>0.05). The *post hoc* analysis showed that metyrapone blocked the disruption of LTM reformation induced by stress administered 1 h after fear memory retrieval (*p*<0.05; Fig. 4B). For stress exposure 12 h after fear memory retrieval, the two-way repeated-measures ANOVA showed significant effects of Test day (*F*_1,20_ = 7.820, *p*<0.05), Stress (*F*_1,20_ = 4.685, *p*<0.05), and Metyrapone (*F*_1,20_ = 4.457, *p*<0.05) and significant Stress X Metyrapone (*F*_1,20_ = 7.720, *p*<0.05) and Test day X Stress X Metyrapone (*F*_1,20_ = 4.906, *p*<0.05) interactions. The *post hoc* analysis showed that metyrapone blocked the disruption of LTM persistence induced by stress administered 12 h after fear memory retrieval (*p*<0.05; Fig. 4C). These results indicate that the disruption of LTM restabilization and persistence induced by stress after fear memory reactivation requires corticosterone synthesis.

## Discussion

In the present study, we demonstrated the differential effects of stress at different time points after learning or memory retrieval on memory performance. Delayed stress or corticosterone administration after fear learning selectively promoted the persistence of LTM. Delayed stress or corticosterone administration after fear memory reactivation selectively destroyed the persistence of LTM. The effects of stress during the late phase after fear learning or fear memory reactivation both required corticosterone synthesis.

The effects of stress on memory are complex. Many factors, such as the emotional valence of the memory, interval between learning and stress, and predominant hormone, might affect the effects of stress on memory [29], [42], [43]. The effects induced by stress have been shown to depend on the memory stage when the subject is exposed to stress [44]. Stress exposure before learning or acquisition can disrupt or enhance memory [45]–[48]. Memory retention performance can be reduced if stress exposure occurs shortly before the memory test [49]–[51]. Studies of the effects of stress have focused on the consolidation of normal or emotional memories and found an inverse U-shaped dose-response relationship with consolidation [52]–[54]. Only a few studies have investigated the role of stress after memory retrieval, suggesting effects of stress on the reconsolidation or extinction process [30], [37], [55]. Animal and human studies have both indicated that stress exposure during the consolidation or reconsolidation process can affect LTM performance, mostly tested 24 h after learning or retrieval, which was consistent with the present results. Interestingly, our study found that stress during the late phase after fear conditioning or fear retrieval selectively affected the persistence of LTM.

The persistence of LTM has been shown to depend on late-phase protein synthesis-dependent processes in the dorsal hippocampus, which was referred to as late consolidation because of its consolidation-like features [23], [24], [27], [28], [41], [56]. Our results showed that stress during the late consolidation time window enhanced the persistence of LTM, without affecting performance in the LTM retention test the next day, which was considered to be the formation of LTM. Consolidated memory can be reactivated to undergo reconsolidation process [11], [13]–[15]. Reconsolidation engages several molecular signaling pathways that are the same as those that are activated during the consolidation process, but an independent mechanism has also been identified [11], [12]. Similar to consolidation, reconsolidation also requires new protein synthesis and has a limited time window of approximately several hours after memory reactivation. Our previous results showed that the restabilized LTM after retrieval also requires a late-phase protein synthesis process after memory reactivation (unpublished data). In the present study, we demonstrated that stress during late reconsolidation disrupted the persistence of restabilized LTM after retrieval but left LTM reformation intact.

Previous research concluded that stress distinctly affects emotional memory consolidation and reconsolidation, improving consolidation but disrupting reconsolidation [29], [30], [35], [44], [57]–[59], which is consistent with our results. The present results showed that stress during the late phase of consolidation and reconsolidation also has different effects, facilitating late consolidation but disrupting late reconsolidation. Below are possible explanations for the distinct effects of stress on late consolidation and late reconsolidation.

### Molecular signaling pathway

Since the discovery of the late consolidation phenomenon, many molecular signaling pathways in the dorsal hippocampus have been shown to be engaged in this process [23], [56]. Brain-derived neurotrophic factor (BNDF), mitogen-activated protein kinases (MAPKs), ERK1/2, zif268, and Fos were significantly increased during the late consolidation window and shown to play critical roles in late consolidation [24], [27], [28], [56]. A molecular mechanism was proposed that was different from the one proposed for consolidation and involved BDNF but not zif268, whereas reconsolidation involved zif268 but not BNDF [12]. These independent mechanisms do not appear to fit for late consolidation and late reconsolidation because BDNF and zif268 were both shown to be crucial for late consolidation [23], [27]. To date, the mechanism that underlies late reconsolidation is unclear. Future studies need to elucidate the molecular mechanisms that underlie the difference between late consolidation and late reconsolidation and the distinct effects of stress on these two memory processes.

### Stress hormones

Glucocorticoids and norepinephrine are the two main stress hormones that underlie the effects of stress on memory. Corticosterone can mimic the effects of stress on both consolidation and reconsolidation, which can be blocked by a glucocorticoid receptor inhibitor or β-adrenoceptor blocker [29], [40], [60]. A recent study showed that the β-adrenergic antagonist propranolol blocked the effect of stress on late consolidation in an inhibitory avoidance task, indicating a critical role for the adrenergic system in late consolidation [41]. In the present study, we found that corticosterone administration during late consolidation or late reconsolidation exerted stress-like effects on the persistence of LTM. Moreover, the effects of stress on late consolidation and late reconsolidation were blocked by a corticosterone synthesis inhibitor. Our results indicate that the effects of stress on late consolidation and late reconsolidation depend on the glucocorticoid system. However, the involvement of concurrent glucocorticoid and noradrenergic activity in the effects of stress on memory does not appear to explain the differential effects of stress on late consolidation and reconsolidation.

### Stress-targeted brain areas

The main brain areas that underlie the effects of stress on memory include the basolateral amygdala (BLA), dorsal hippocampus, and prefrontal cortex (PFC) [40], [58], [61]–[63]. Stress-induced glucocorticoid and noradrenergic activation in the BLA can directly affect memory in the BLA or modulate memory processes in the dorsal hippocampus and PFC [40], [62]. Previous studies indicated that the dorsal hippocampus was the main brain area recruited during late consolidation [23], [56], and stress-induced BLA stress system activation may indirectly regulate the memory process in the dorsal hippocampus. Stress can also directly affect the late consolidation process in the dorsal hippocampus. Direct infusion of norepinephrine into the dorsal hippocampus during late consolidation enhanced the persistence of LTM [28]. Although no direct evidence has demonstrated the role of the hippocampal glucocorticoid system in late consolidation, several studies have identified a critical role for hippocampal glucocorticoids in other memory stages [64], [65]. The present results cannot elucidate the precise role of the stress systems in the BLA and dorsal hippocampus during late consolidation or late reconsolidation on the persistence of LTM. Future studies should reveal such mechanisms.

In summary, we found that stress during the late phase after fear learning or retrieval selectively affected the persistence of LTM. Stress and corticosterone administration improved the late consolidation of memory but disrupted late reconsolidation. The effects of stress on late consolidation and late reconsolidation were dependent on corticosterone synthesis. These results indicate that the corticosterone system participates in the persistence of LTM after both acquisition and retrieval.

## Materials and Methods

### Subjects

Male Sprague Dawley rats, weighing 260–280 g upon arrival, were obtained from the Laboratory Animal Center, Peking University Health Science Center. They were housed in groups of five in a temperature (23±2°C)- and humidity (50±5%)-controlled animal facility and were maintained on a 12 h/12 h light/dark cycle with *ad libitum* access to food and water. All of the experimental procedures were performed in accordance with the National Institutes of Health *Guide for the Care and Use of Laboratory Animals* and were approved by Biomedical Ethics Committee of Peking University of animal use and protection.

### Drugs

Corticosterone and the corticosterone synthesis inhibitor metyrapone were purchased from Sigma (St. Louis, MO, USA). Corticosterone was dissolved in saline that contained 2% ethanol, and metyrapone was dissolved in a vehicle that contained 40% polyethylene glycol and 60% saline to reach an appropriate concentration. All of the drugs were freshly prepared before the experiments. The doses of the drugs (10 mg/kg corticosterone, i.p.; 40 mg/kg metyrapone, i.p.) were based on our previous report [30]. Metyrapone was administered 40 min before stress [30].

### Contextual fear memory

Contextual fear conditioning was conducted in four identical isolated shock chambers (Shanghai Jiliang Software Technology Co. Ltd, Shanghai, China) that were used in our previous study [66]. The contextual fear conditioning procedure was modified from previous studies [67]. The rats were handled for 3 days before conditioning. On the day of the experiments, they were placed into the conditioning chamber and allowed to explore the chamber for 2 min, after which they received an electric footshock (1 s). The shock intensity was 0.4 mA for weak training and 1.0 mA for strong training. The 2 min/1 s procedure was repeated a total of three times, and the rats were allowed to explore the conditioning chamber for an additional 1 min. After removing the rat from the chamber, the chamber was cleaned with 75% alcohol to eliminate any residual odor. To reactivate memory (i.e., retrieval), the rats were exposed to the conditioning chamber for 3 min without shock. Memory was tested by exposing the rats to the conditioning chamber for 5 min without shock. All of the experimental sessions were video-recorded for offline analysis. Freezing behavior was defined as the lack of all movement, with the exception of respiration.

### Cold swim stress

The cold swim stress protocol was adapted from our previous study [30]. Briefly, the rats were placed for 5 min into a 35 cm diameter ×40 cm height plastic cylinder that was filled to a depth of 30 cm with ice-cold water [30], [68]. The rats were then removed, dried, and returned to their home cage.

### Statistical analysis

The data are expressed as mean ± SEM. The statistical analyses were performed using repeat-measures ANOVA for all of the experiments. The within-subjects and between-subjects factors are stated in the Results section. *Post hoc* analyses of significant effects in the ANOVAs were performed using the Least Significant Difference test. Values of *p*<0.05 were considered statistically significant.

### Special Experiment

#### Experiment 1: Effect of stress and corticosterone at different time points after fear conditioning on LTM

We first tested whether stress or corticosterone administration at different time points after conditioning would distinctly affect LTM. Weak training (0.4 mA, 1 s, three shocks) was performed in this experiment. Three groups of rats were separately exposed to forced swim stress 1, 12, or 24 h after contextual fear conditioning. A group of rats without stress was used as a control. To test whether corticosterone affects memory similarly to forced swim stress, three groups of rats were separately administered corticosterone 1, 12, or 24 h after contextual fear conditioning. An additional group of rats that did not receive these treatments were used as a control. Memory was tested 2 and 7 days after fear conditioning (*n* = 6–9 per group).

#### Experiment 2: Effect of corticosterone synthesis inhibitor on enhancement of LTM induced by stress after fear conditioning

We tested whether a corticosterone synthesis inhibitor would affect the post-conditioning stress induced enhancement of LTM formation or persistence. Weak training (0.4 mA, 1 s, three shocks) was performed in this experiment. Eight groups of rats were separately administered the corticosterone synthesis inhibitor metyrapone 40 min before exposure to forced swim stress, which was given 1 or 12 h after fear conditioning. Memory was tested 2 and 7 days after memory reactivation (*n* = 6 per group).

#### Experiment 3: Effect of stress and corticosterone at different time points after fear memory reactivation

We then tested whether stress or corticosterone at different time points after memory reactivation distinctly affect recent memory. Strong training (1.0 mA, 1 s, three shocks) was performed in this experiment. Two days after fear conditioning, three groups of rats were separately exposed to forced swim stress 1, 12, or 24 h after memory reactivation. A group of rats that were not exposed to stress after memory reactivation was used as a control. To test whether corticosterone affects reactivated memory similarly to forced swim stress, three groups of rats were separately administered corticosterone 1, 12, or 24 h after contextual fear reactivation. An additional group of rats that did not receive these treatments was used as a control. Memory was tested 2 and 7 days after memory reactivation (*n* = 6–8 per group).

#### Experiment 4: Effect of corticosterone synthesis inhibitor on disruption of LTM induced by stress after fear memory reactivation

We tested whether a corticosterone synthesis inhibitor would affect the post-retrieval stress induced disruption of LTM restablization or persistence. Strong training (1.0 mA, 1 s, three shocks) was performed in this experiment. Eight groups of rats were separately administered the corticosterone synthesis inhibitor metyrapone 40 min before exposure to forced swim stress, which was given 1 or 12 h after fear memory reactivation. Memory was tested 2 and 7 days after memory reactivation (*n* = 6 per group).
